# Impact of #PsychTwitter in promoting global psychiatry: A hashtag analysis study

**DOI:** 10.3389/fpubh.2023.1065368

**Published:** 2023-02-22

**Authors:** Faisal A. Nawaz, Mehr Muhamad Adeel Riaz, Christos Tsagkaris, Umme H. Faisal, Elisabeth Klager, Maria Kletecka-Pulker, Oliver Kimberger, Harald Willschke, Nagina Khan, Meshal A. Sultan, Atanas G. Atanasov

**Affiliations:** ^1^Department of Psychiatry, Al Amal Psychiatric Hospital, Dubai, United Arab Emirates; ^2^Department of Psychiatry and Behavioral Sciences, Faisalabad Medical University, Faisalabad, Pakistan; ^3^European Student Think Tank, Public Health and Policy Working Group, Amsterdam, Netherlands; ^4^All India Institute of Medical Sciences, Kalyani, India; ^5^Ludwig Boltzmann Institute for Digital Health and Patient Safety, Medical University of Vienna, Vienna, Austria; ^6^Institute for Ethics and Law in Medicine, University of Vienna, Vienna, Austria; ^7^Department of Anaesthesia, Intensive Care Medicine and Pain Medicine, Medical University of Vienna, Vienna, Austria; ^8^Center for Addiction and Mental Health, Toronto, ON, Canada; ^9^Department of Osteopathic Medicine, Touro University Nevada, Henderson, NV, United States; ^10^Mental Health Center of Excellence, Al Jalila Children's Specialty Hospital, Dubai, United Arab Emirates; ^11^College of Medicine, Mohammed Bin Rashid University of Medicine and Health Sciences, Dubai, United Arab Emirates; ^12^Institute of Genetics and Animal Biotechnology of the Polish Academy of Sciences, Jastrzȩbiec, Poland

**Keywords:** social media, psychiatry, Twitter, PsychTwitter, hashtag

## Abstract

**Introduction:**

Multiple studies have shown how valuable Twitter hashtags can be for promoting content related to different themes in the online community. This arena has grown into a rich data source for public health observation and understanding key trends in healthcare on a global scale. In the field of mental health in particular, it would be of benefit to understand and report the key stakeholders' (individual mental health professionals, academic organizations and their countries) trends and patterns of psychiatric knowledge and information dissemination using #PsychTwitter.

**Objective:**

In this study, we aim to evaluate the achieved outreach of psychiatry-related tweets using the hashtag #PsychTwitter.

**Methods:**

We utilized the Symplur Signals research analytics tool to characterize tweets containing #PsychTwitter from the 20th of August, 2019, to the 20th of August, 2022.

**Results:**

The #PsychTwitter movement resulted in 125,297 tweets that were shared by 40,058 Twitter users and generated a total of 492,565,230 impressions (views). The three largest identified groups of contributors were Doctors (13.8% of all tweets), Org. Advocacy (6.2% of all tweets), and Researcher/Academic (4% of all tweets) stakeholders. The top influential accounts consisted of 55 psychiatrists and 16 institutional or organizational accounts. The top 5 countries from where most of the tweets containing #PsychTwitter were shared include the United States (54.3% of all users), the United Kingdom (10.4% of all users), Canada (4.9% of all users), India (2% of all users), and Australia (1.8% of all users).

**Conclusion:**

This is the first of its kind study featuring the influence and usage of #PsychTwitter and covering its global impact in the field of psychiatry using the Twitter platform. Our results indicate that Twitter represents a broadly used platform for mental health-related discussions.

## Introduction

In the past decade, social media has greatly influenced both personal and professional lives (1). Social media apps help doctors to stay updated with the advances in medical science by facilitating easy consultation, collaboration, and communication with et al. from different parts of the world (2, 3). Various social networking sites, such as Facebook, LinkedIn, Twitter, and YouTube, cater to different audiences *via* different approaches, functions, and utilities. The majority of Twitter data is in the public domain. This arena has grown into a rich data source for public health observation and understanding key trends in healthcare communications on a global scale.

Twitter is a free-to-use, open-access social networking and micro-blogging site that allows registered users to post, read and share short messages called tweets. Users can post images, short videos, or website URLs with their followers, while there is 280-character restriction on tweets (up from 140) (4). Twitter utilizes a feature called “hashtag” which enables users to easily connect posts of a specific topic under an “umbrella” designated by a name, such as #PsychTwitter. This hashtag enables users to easily search and filter tweets pertaining to psychiatry on Twitter (5).

Multiple studies have shown how valuable hashtags can be for promoting material related to different themes and events such as disease-specific tweets (6–8), meeting or conference-related content (9–11), and Twitter-based chats and journal clubs (12). Moreover, it is pertinent to mention that academia has widely reported the social media usage trends, the effects of social media use on mental health and well-being, and the possibility to exploit social media's accessibility and interactive content to improve the delivery of interventions for health and mental healthcare (13). For example, one of the relevant scholarly articles incorporated the use of the keywords “mental health”, “mental illness” and “social media” to evaluate the potential role of social media as an intervention platform for providing support to people with mental disorders and strengthening current mental health services (13). Another study reported the trends of social media usage among physicians, whereby 65% of the physicians reported using the space for personal and professional purposes (14). However, both of the mentioned important studies did not study the role or usage trends of Twitter by mental health professionals using global psychiatric discussion spaces on Twitter using #PsychTwitter, and up to know the usage of this hashtag has not been a subject to detailed longitudinal analysis.

As evident from the COVID-19 infodemic parallel pandemic, it is an established that social media can greatly influence the decision-making power of the general population when it comes to scientific information dissemination (15). In this context, it is especially important to analyze and report this area of the global psychiatry-related discussions. Furthermore, there is a knowledge gap who uses that kind of social media space, especially in regard of Twitter users disseminating information using #PsychTwitter. In this study, we aim to evaluate the achieved outreach (defined as the act of engaging of the Twitter community, measured by engagement metrics like the number of impressions and tweets, which were used as the key performance indicators) of psychiatry-related tweets using the hashtag #PsychTwitter.

## Methods

### Hashtag development and outreach

This study observed the growth of #PsychTwitter over a 3-year period, ranging from the 20th of August, 2019, to the 20th of August, 2022. The initial registration of #PsychTwitter was established as part of the Symplur healthcare hashtag project (16). The nature of this hashtag was predominantly aimed at connecting psychiatry and the wider mental health community through posts in the form of “tweets” on topics of education, research, resources, events, or opportunities associated with advancing mental healthcare. Other forms of hashtag engagement included retweeting content, commenting on tweets, and community participation in live discussions. A Twitter list was also identified as part of promoting #PsychTwitter, which included 46 members and 550 follower accounts of individuals and organizations which were focused on actively sharing content related to psychiatry and mental health, thus amplifying the hashtag visibility (17).

### Data extraction and analysis

We utilized the Symplur Signals research analytics tool to characterize of tweets containing #PsychTwitter from the 20th of August, 2019, to the 20th of August, 2022.

Symplur Signals is a comprehensive hashtag analysis tool that enables long-term tracking of tweets that contain focused hashtags pre-registered with the Symplur healthcare hashtag project (16). The analysis conducted with Symplur Signals evaluated the total number of tweets (including retweets), impressions (i.e., views of tweets), and unique users disseminating tweets containing #PsychTwitter (including user categorization to specific healthcare stakeholder groups). All tweets containing the hashtag #PsychTwitter were analyzed with Symplur Signals, without any restrictions on language, location of users, or other parameters. The most shared tweet, article, poll, image, and video clip were also identified. The collective data was then extracted to Microsoft Excel for final interpretation. Primary outcome measures for the achieved outreach (defined as the act of reaching out to the Twitter community) and awareness (defined as bringing relevant information and knowledge to the Twitter community) were the number of tweets and impressions. Furthermore, this analysis also included parameters such as the content of these tweets (e.g., links, mentions of other accounts, images), major influencers, tweet languages, sentiment analysis, and geolocation trends. The Healthcare Social Graph Score is determined by tracking over 35,000 healthcare topics in real-time on Twitter. Based on the tracking, a top impact profile list for the past year for each of the 35,000 topics is generated. The rankings are created using the impact algorithm SymplurRank. In addition to these rankings, Symplur also measures the quality of the conversations for every topic each week. This conversation quality score is then factored with conversation volume to provide a weighted measure for the impact scores. Finally, the 52 weekly rankings and quality scores are combined into a single number for each social media profile and then normalized on a scale of 0 to 100. This final number is known as the Healthcare Social Graph Score (18, 19).

Additionally, the SymplurRank algorithm was used to measure the influence of specific accounts and the importance of the content that they share for selected datasets and parameters. The algorithm ranks influential accounts by measuring the number of quality mentions received, whereby the quality of a mention is determined by the account that gave the mention, and the influencer account's individual impact on the topic along with its healthcare stakeholder status (20). In order to retrieve the individual characteristics of these influential accounts, we evaluated their Twitter biographies and practice or institutional websites. This was accomplished by manually reviewing the list of influencers by visiting their Twitter profile and extracting details on gender, profession, and type of account created (institutional account/personal account) for further analysis.

Since commercial software (Symplur Signals) was used to perform the analysis, further details about the specific statistical techniques used by Symplur to generate several of the variables quantified (e.g., influencers, sentiment scores, user categories etc.) are not available.

### Ethical approval and informed consent

This study is exempted from research ethics review since it is based on pre-existing publicly available data and did not involve the prospective collection of data from human participants. All presented data are anonymized and the study does not state any information related to specific Twitter user accounts.

## Results

**Figure 1:**
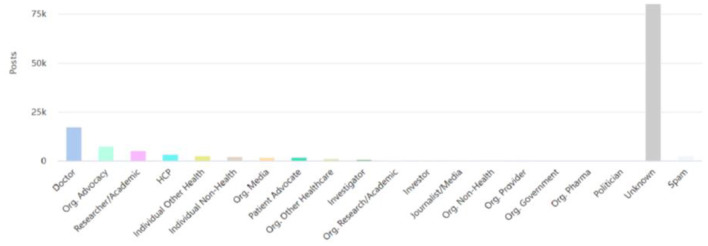
Number of tweets shared by major healthcare stakeholders.

Across a 3-year period of hashtag analysis, the #PsychTwitter movement resulted in 125,297 tweets (including 75,548 retweets) that were shared by 40,058 Twitter users and generated a total of 492,565,230 impressions (views). Accounting for the percentage distribution of #PsychTwitter-posting users in various healthcare stakeholders categories (data derived from Symplur Signals, with the classification being based on information provided in the Twitter biographies of the users), the three largest identified groups of contributors were Doctors (13.8% of all tweets), Org. Advocacy (6.2% of all tweets), and Researcher/Academic (4% of all tweets) stakeholders. The complete distribution of the 20 identified categories is depicted in Figure 1 (note: 65.1% of the tweets were from accounts that did not provide sufficient information to be categorized, and were labeled as “Unknown”).

The top 5 countries from where most of the tweets containing #PsychTwitter were shared (based on the locations at which the posting accounts were registered) include the United States (54.3% of all users), the United Kingdom (10.4% of all users), Canada (4.9% of all users), India (2% of all users), and Australia (1.8% of all users). Table 1 represents the regional ranking of users that tweeted using #PsychTwitter.

**Table 1:** Regional popularity of #PsychTwitter.

**Sr No**.	**Country**	**Users**	**Percentage (%)**
1	United States of America	21,735	54.3
2	United Kingdom	4,158	10.4
3	Canada	1,961	4.9
4	India	796	2
5	Australia	741	1.8

The most commonly co-occurring hashtags with #PsychTwitter include #MedTwitter, #MentalHealth, #Psychiatry, and #MedEd. The top 100 influencer accounts were analyzed according to the SymplurRank algorithm in relation to their impact using #PsychTwitter. According to the algorithm, the top #PsychTwitter influencer accounts consisted of 55 psychiatrists and 16 institutional or organizational accounts. The remaining accounts included those of psychologists, psychiatry educational communities, journals, and trainees in psychiatry. Among the top 100 accounts, 31 consisted of female psychiatrists and 24 were male psychiatrists. Interestingly, all of the top 20 psychiatrists (with Symplur Rank ranging from 100 to 43.69) using #PsychTwitter were based in the USA, with one single exception (one was based in Canada instead).

Sentiment analysis of the relevant tweets shared in the study period was also performed with Symplur Signals and revealed an average of 53.9% positive and 46.1% negative sentiment. Qualitative evaluation of the positive-sentiment tweets revealed that they often expressed gratitude (“thank you”, “gratitude”, “thanks to some great mentors”, “many thanks”, “gracias”, etc.), wishing all best (“my best to you all”, “wish you a joyful day”, “wish you all a blessed day”, “wish you all a wonderful weekend”, “wish you all a beautiful day”, etc.), and appreciation (“so awesome”, “amazing”, “so welcoming”, “source of joy”, “really amazed”, etc.) while negative-sentiment tweets often referred to diseases or health conditions (“mental illness”, “severe depression”, “pandemic”, “mental health problems”, “depression”, etc.), harmful actions (“self-harm”, “abused”, “abandoned”, “harm”, “stigmatized”, etc.), negative mental states (“grief”, “psychological pain”, “mourning”, “shame”, “pain”, etc.), and end of life (“suicide”, “death”, “American suicides”, “suicide attempts”, “risk of suicide”, etc.).

The tweet that induced most engagements (371 engagements) was a call from a medical doctor encouraging physicians to invest more efforts to understand specific patients illnesses in order to achieve superior treatments. The most shared article (posted 163 times) was a 2020 study published in Pediatrics entitled “Pubertal Suppression for Transgender Youth and Risk of Suicidal Ideation” (21). Meanwhile, the Twitter poll that received most votes (219 votes) was posted by psychiatrist who asked the users if they have ever heard someone to equate diverse representation with lack of merit. The most shared visual (image; 522 shares) was the cover-page of the “Diagnostic and Statistical Manual of Mental Disorders, Fifth Edition, Text Revision (DSM-5-TR)”, by American Psychiatric Association Publishing (22). Concerning the most shared video clip (200 shares), it represented a call by German and Austrian physicians and patients for more research to be conducted on myalgic encephalomyelitis/chronic fatigue syndrome.

The top 3 institutional or organizational accounts included the American Psychiatric Association, the American Academy of Child and Adolescent Psychiatry, and the Association of Directors of Medical Student Education in Psychiatry.

## Discussion

We carried out a three-year longitudinal examination of the activity, users, and content associated with #PsychTwitter from the 20th of August 2019 to the 20th of August 2022; using the Symplur Signals hashtag analytics tool. The cumulative use of #PsychTwitter yielded 125,297 tweets that were shared by 40,058 Twitter users and generated a total of 492,565,230 impressions (views) from different geographical locations. The majority of users only sent one English-language tweet throughout this time. To make tweets easier to comprehend, links, mentions of other accounts, and graphics were frequently added. The vast majority of assembled content that includes references to other accounts and connections to external sources served as proof of the knowledge-sharing that occurred during this campaign. Current techniques for social media analysis such as data mining and sentiment analysis have shown promising use-cases for research. Despite the early stage of positive outcomes, a number of challenges remain unaddressed in this context. Common issues with data mining include extracting and filtering through large quantities for data, which is often dynamic and complex in nature. The dynamic nature of data mining on social media includes both structured (texts) and unstructured (images, videos, live events) forms of communication across a variety of social media platforms (23). Furthermore, sentimental analysis relies on emotion detection between overlapping content shared online, which can be challenging due to difficulty in detection of sarcastic terms, negation handling, spam accounts postings, and lack of training data sets for machine learning algorithms analyzing non-English content (24). Healthcare data on social media adds to an additional layer of specificity which was seen for example in a study by Salud et al. that found challenges with supervised learning and lexicon-based sentimental analysis in analyzing content on drug-based reviews by patient communities that often incorporate complex medical terminologies, as compared to analyzing physician-reviews online that used relatively simpler terms online (25). This highlights the need for developing specialized sentiment analysis tools that are focused on detecting medical terminologies on social media in order to avoid gaps in data consistency and completeness of research. The top twenty influencers were analyzed according to SymplurRank, Healthcare Social Graph Score, number of mentions, number of shared tweets containing #PsychTwitter, and a total number of generated impressions (views) of the respective tweets. As depicted in Figure 1 majority of the tweets using #PsychTwitter were posted by doctors followed by advocacy organizations and researchers. There has been an increase in the trend of using Twitter as an active platform to create awareness and disseminate information about various conditions in the psychiatric field. Notably, “Patient Advocate” was the eight most prevalent stakeholder-group that shared #PsychTwitter containing tweets, and it is a reasonable assumption that this group might contain some users who are current or former patients or patient relatives who are qualified to bring into the discussion unique patient-derived perspectives.

There has been active evidencereporting since 2014, that examines the discourse of psychiatry on Twitter (26). Literature shows social media microblogging sites like Twitter have played an important role in highlighting mental health conditions. The general Twitter users concentrated on a small number of phrases related to psychiatric disorders like autism, schizophrenia, and depression. The hashtag #MDLL (#mydepressionlookslike) was created by Lachmar et al. that analyzed 3,225 tweets to highlight themes that come up frequently when people on Twitter discuss depression ranging from dysfunctional thoughts to the impact on social life and social support-seeking behaviors (27). Reavley et al. analyzed tweets posted in the English language that discussed the topics of schizophrenia and depression (28). Using the hashtags #schizophrenia and #depression, the studies explored patterns associated with the inclusion of these hashtags in research.

This project further explores the demographical variations associated with #PsychTwitter users in regard to various aspects, such as gender, an individual vs. an organization, and psychiatric/academic background. United States (54.3% of all users)-based #PsychTwitter users were among the top accounts using the hashtag, followed by the United Kingdom (10.4% of all users), Canada (4.9% of all users), India (2% of all users), and Australia (1.8% of all users) to make the top five countries. It has been reported that physicians across the United States advocate actively on social media about various health promotion campaigns (29). During the course of the 5-year period, the number of physicians in the United States using Twitter has more than doubled (up by 112%) (29). The most popular topics included general health, medical education, and mental health during the examined period. It is noteworthy to mention that India is the only developing country in the top identified regions, possibly due to increased social media awareness and accessibility in the general population and psychiatrists. The past few years have seen an increase in the trend of internet access to social media usage by low and middle-income countries (30). Recent data shows that the United States tops the Twitter user number 76.9 million people followed by India at 23.6 million active Twitter users (31).

The data reports that top #PsychTwitter influencer accounts are dominated by psychiatrists because they are the core of the #PsychTwitter community. However, Udayakumar et al. analyzed previously the demographics of Twitter users in the field of psychology and reported that 31.4% of the accounts that used psychology terms belonged to the field of academia with only 16.5% having filed expert background (32). A recent study shows that 87.9% of the healthcare providers who participated in the study denoted that they use social media and encouraged their patients to research clinical conditions on social media (33). It is important to mention that the healthcare workers who advocated the use of social media were under 40 years of age. Furthermore, research indicates that 41% of American healthcare consumers used social media to select their healthcare providers (34). Additionally, 26% of American hospitals presently use social media in some capacity (35). Healthcare providers reported that social networking has given healthcare professionals a tool to overcome obstacles in patient care delivery.

If we analyze the #PsychTwitter algorithm through the gender lens among the top 100 accounts, 31 belonged to female psychiatrists as compared to the 24 male psychiatrists' accounts. The observed gender ratio is different compare with the data of Udayakumar et al. that showed 56% of males posted psychology-related tweets as compared to 37.5% of female users (32). When we looked into the types of the top 3 institutional or organizational accounts, the American Psychiatric Association, the American Academy of Child and Adolescent Psychiatry, and the Association of Directors of Medical Student Education in Psychiatry were among the #PsychTwitter most often posting accounts. Literature suggested that social media is one of the reliable methods to design, collect, implement and get feedback for advocacy works and physician-patient two-way communication (36). The above-mentioned analysis shows the influence of advocacy and mental health awareness that is bringing together different influencers engaging on Twitter between themselves and with organizational accounts in the psychiatry community by using #PsychTwitter. It brings together different influencers engaging on Twitter between themselves and with organizational accounts. In a similar line of communication, Twitter public policy team launched a campaign in 2020 amid COVID-19 pandemic in collaboration with 60 mental health partners (37). The campaign tagline was quoted 170,000 times engaging more than half a million users and 70 global mental health organizations by using #MentalHealthAwarness, #LetsTalk, and #TogetherWeCan. However, this campaign did not report the specific mention and usage of #PsychTwitter.

### Limitations and future research

This study is subject to a number of limitations regarding the methods and the extent of the analysis. The general challenge of detecting emotional contexts of social media content shared is pertinent to this study as well. Thus, despite the significance of emotion detection in the mental health field, the sentiment analysis performed within this study is limited in assessing the complete spectrum of emotional states, as well as might not well reflect the complex medical terminologies shared within the psychiatry community. Furthermore, tweets shared from spam accounts may have had some influence on quantified the impacts (likes, comments, retweets) of this hashtag, despite the comparably small share of spam accounts identified (Figure 1). Envisaging the limited information provided in biographies of Twitter users, some important demographic data (e.g., gender and age) could not be analyzed. Previous studies have investigated different fields of biomedical science and practice on social media by analyzing the entire content of one or more social media platforms for a number of keywords associated with the topic under question. For instance, a study by Sahu et al. (38) explored the hashtag #Orthotwitter together with tweets including terms such as orthopedic surgery or orthopedics. Considering that tweets related to psychiatry and mental health did not necessarily use the hashtag #PsychTwitter, it is possible that content relevant to the subject of the search remained unidentified (10). This challenge also limits the identification of non-English content shared within the online mental health community, providing that respective tweets did not include #PsychTwitter.

At a broader level, a study by Kawchuk et al. (39) analyzed misinformation related to the effects of chiropractic manipulation on immunity by means of a social media searching software. Studies with a broader scope than a particular hashtag were, hence, more likely to capture the entire spectrum of content related to the field under investigation. Notwithstanding this limitation, the orientation of the present study toward the hashtag #PsychTwitter is in line with emerging knowledge about the role that Twitter hashtags can play in sharing resources and spreading awareness among healthcare professionals (40, 41). Similarly, the study examined Twitter only, although hashtags are also used on other popular social media platforms such as Facebook (Meta) and Instagram. Theoretically, this constitutes an additional limitation to the content that was subjected to further analysis, since Twitter users familiar with #PsychTwitter may reshare their tweets on other platforms. Nonetheless, the use of a hashtag containing the term “Twitter” is less likely to happen intentionally on social media platforms other than Twitter. Moreover, narrowing the present study down to Twitter enabled the authors to weigh on previous studies describing the particular features of Twitter interactions in biomedical discourse. This includes the active representation of biomedical journals and publishers apart from individual researchers and physicians and the rising awareness of Twitter-specific metrics of awareness and activities among the biomedical community (42, 43). It is worth noting that, although Symplur Signals has been extensively used for analyzing the impact of hashtags among social media communities, to our knowledge, no peer-reviewed study has been conducted to assess the validity of Symplur Signals techniques for content analysis. This adds as a limitation to our study with regards to handling of missing data and relying on proprietary algorithms that are unvalidated externally. Future studies should explore the use of such platforms as standardized tools for hashtag analysis. The scope of this study was also limited by barriers with transparency in obtaining data through the Symplur Signals platform, which does not elaborate on the statistical techniques used for content analysis. Future studies should also take into account the limitation of social metrics such as tweets and impressions not entirely reflecting the discussions, level of understanding of participants and “readability” of tweets by users. This may lead to over-reporting of data results if not accounted.

Future research can contribute to tackling the aforementioned obstacles and strengthening the understanding of online discourse. Within Twitter, it would be interesting to position the analysis of #PsychTwitter in the broader image of psychiatry and mental health content in the same medium. Comparing the outreach and the awareness achieved by tweets containing the hashtag and tweets not containing it can provide more evidence regarding the influence of hashtags. If hashtags prove more influential than general, unlabeled content on the matter, health promoters in the field can be recommended to include the use of #PsychTwitter and its co-occurring hashtags in their outreach strategies. Furthermore, numerous entities and individuals maintain a presence on more than one social media platform. Therefore, it is important to examine whether the same or similar hashtags and subsequent communities have been established on different social media platforms. Comparing the audience and the outreach of each of them could determine which medium is more suitable for communication between peers and for raising awareness among the public.

## Conclusion

This is the first of its kind study featuring the influence and usage of #PsychTwitter and covering its global impact in the field of psychiatry that allowed the assessment of a large sample of tweets and views (impressions) over a 3-year period for longitudinal analysis. Our conclusions emphasize the importance of professional voices in the exchange of accurate healthcare information during the recent COVID-19 pandemic. Our results indicate that social media microblogging sites like Twitter are broadly used for mental health-related discussions. In addition, it is necessary for future research to develop knowledge with a focus on the deeper and wider context of psychiatric diagnosis, care plans, and pathways that could be collected through online data. Special attention should be given to the topic and content of hashtag exchange in order to evaluate the impact of misinformation related to mental health on social media platforms.

## Data availability statement

The original contributions presented in the study are included in the article/supplementary material, further inquiries can be directed to the corresponding author.

## Author contributions

FN, MR, CT, UF, and NK prepared the first draft of this manuscript. AA analyzed results. FN, MR, CT, UF, EK, MK-P, OK, HW, MS, NK, and AA reviewed, edited, and approved the final manuscript. All authors contributed to the article and approved the submitted version.
